# A Novel Technique of Mixed Reality Systems in the Treatment of Spinal Cord Tumors

**DOI:** 10.7759/cureus.23096

**Published:** 2022-03-12

**Authors:** Ryoma Aoyama, Ukei Anazawa, Hiraku Hotta, Itsuo Watanabe, Yuichiro Takahashi, Shogo Matsumoto

**Affiliations:** 1 Orthopaedics, Tokyo Dental College Ichikawa General Hospital, Ichikawa, JPN

**Keywords:** intraoperative support, stereoscopic images, holoeyes, preoperative planning, mixed reality, spinal cord tumor

## Abstract

Several reports have compared spinal cord tumor removal techniques but none have clearly described the appropriate site and level of indication for laminectomy or laminoplasty. The approach method for tumor removal depends on the type and localization of the tumor and the surgeon's skill. Therefore, a system that can suggest various surgical techniques is useful for spinal cord tumor surgery. The mixed reality system introduced in this paper is an excellent system that can suggest various surgical procedures. Using this system for spinal cord tumor removal, we made the surgery less invasive; therefore, we introduced this system and demonstrated its usefulness. Stereoscopic data of the patients with spinal cord tumors were obtained from preoperative myelogram-CT data. Stereoscopic laminectomy models including tumors were created using Blender, a free three-dimensional (3D) image editing software. We observed these data as 3D object images using a head-mounted display (HMD). This HMD is commercially available and relatively inexpensive. The surgical procedure is determined by considering those 3D images, radiological diagnosis, and the skill of surgeons. Intraoperative confirmation of the laminectomy site could be performed using the HMD. The 3D visualization of pathological conditions resulted in correct preoperative surgical planning and less invasive surgery in all five cases. Stereoscopic images using HMDs allow us a more intuitive understanding of the positional relationship between the tumor and spinal structure. These 3D object images can bring us more accurate preoperative planning and proper determination of surgical methods.

## Introduction

Spinal cord tumors are often removed by laminectomy or laminoplasty [1-10]. To prevent postoperative spinal instability, it is important to minimize resection of the spinal component. To minimize damage to the spinal cord, it is necessary to have enough operating space for easy handling of the tumor and spinal cord, which requires wide bony resection. Removing spinal cord tumors requires the surgeons to weigh these conflicting factors against the pathological condition and the surgeon's skill. This dilemma provides a variety of approaches to the tumor, ranging from conventional laminectomy [5] with or without instrumentation to laminoplasty [6,7,9,10]. Less invasive surgeries that could minimize the removal of the bone and soft tissue have the advantages of reduced blood loss, preventing spinal fluid leakage, preventing postoperative deformity, which is especially problematic in children, and shorter hospital stay [1-4,6,7,10]. Instrumentation surgery would be required in many cases treated with extensive joint resection [8].

Plain images such as CT, myelogram-CT, and MRI have been widely used to determine the site and extent of bone resection or laminoplasty. Recent advances in image editing technology have made it easy to display stereoscopic images of bones and nerves on a flat display. However, if a stereoscopic image appears on a conventional flat display, this image is only a two-dimensional (2D) image. It takes time for the evaluators to convert a 2D image to a three-dimensional (3D) one in their brain. On the other hand, advances in mixed reality (MR) technology using head-mounted displays (HMDs) have made it possible to observe stereoscopic images in 3D projected with HMDs [11-14]. There are several reports that the MR systems are effective for tumor removal because surgeons can confirm important organs such as blood vessels hidden in the operation field [14]. In addition, by using the HMD to observe the spinal cord tumor as a 3D image, the surgeons can intuitively grasp the tumor's location before surgery. This observation intuitively determines the optimal decompression method and laminectomy site preoperatively. The system introduced in this paper is a relatively inexpensive commercialized system, and therefore it is easy to operate [11]. We describe the usefulness of this MR system for spinal cord tumor removal.

## Technical report

Applied cases

Case 1

**Figure 1:**
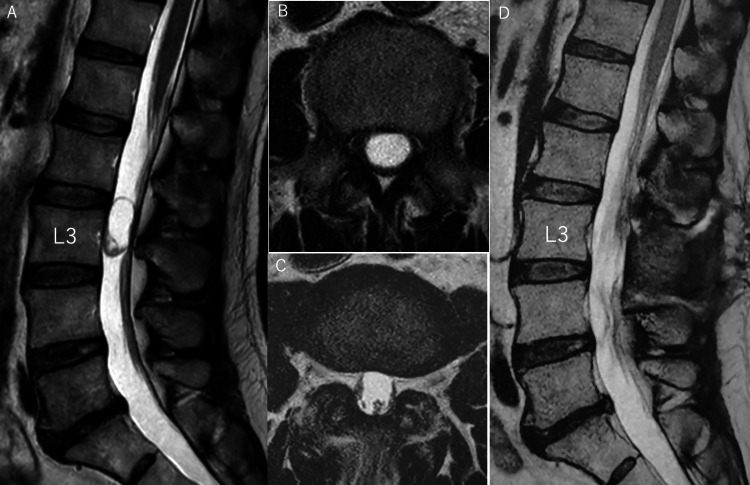
Pre- and postoperative MRI of case 1 (A) Preoperative MRI T2-weighted sagittal section showed the tumor including cysts from the L2/3 disc to the L3 vertebral body. (B) Preoperative MRI T2-weighted cross-sectional image showed that the tumor occupied the entire dural canal. (C) Postoperative MRI T2-weighted cross-sectional image showed no residual tumor. (D) Postoperative MRI T2-weighted sagittal section showed no residual tumor, and the L3 lamina had been removed.

This is a case of a 63-year-old man with L3 schwannoma Preoperative MRI showed a neoplastic lesion including a cyst posterior to the L3 vertebral body. The tumor was removed by L3 laminectomy with the longitudinal split of the L3 spinous process (Figure 1). Postoperatively, the patient's lower limbs pain improved markedly.

Case 2

**Figure 2:**
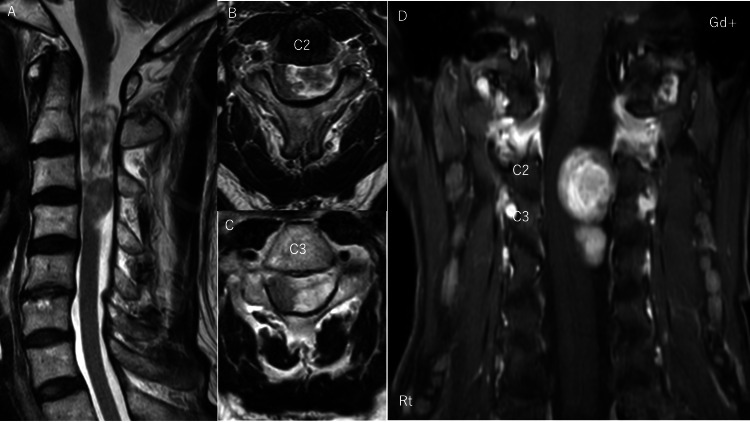
Preoperative MRI of case 2 (A) T2-weighted sagittal section showed the spinal cord tumor from C2 to the C4 vertebral body. (B) T2-weighted cross-sectional image at C2 level showed that the tumor occupied almost all of the dural canal. (C) T2-weighted cross-sectional image at C3 level showed that the tumor occupied more than half of the dural canal. (D) A gadolinium-enhanced coronal section showed that the spinal cord tumor was distributed to the left side.

**Figure 3:**
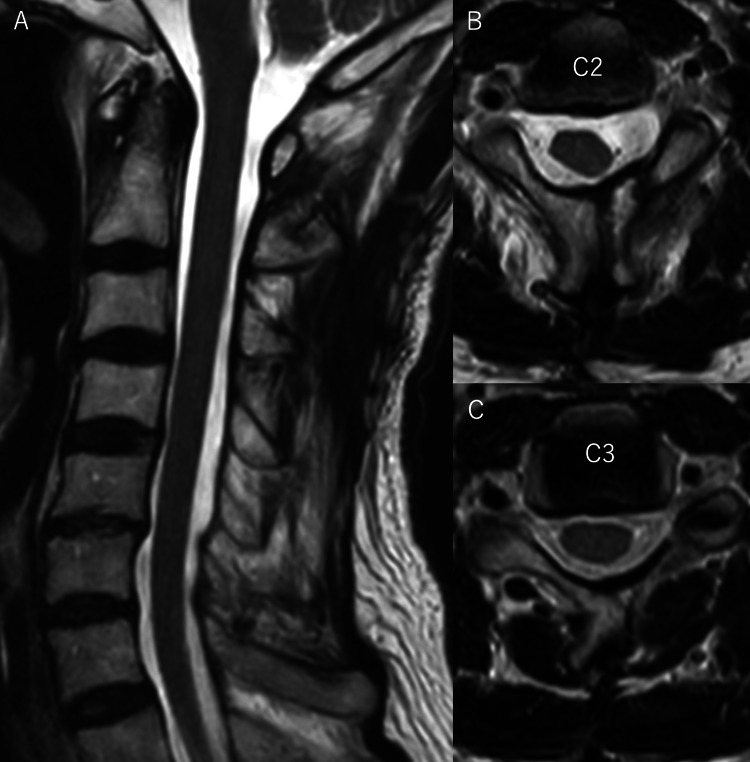
Postoperative MRI of case 2 (A) T2-weighted sagittal section showed that the spinal cord tumor had been completely removed, and the spinal structure had been well preserved. (B) T2-weighted cross-sectional image at C2 level showed that the spinal cord tumor had been completely removed and no muscle damage of C2 had occurred at all. (C) T2-weighted cross-sectional image at C3 level showed that the spinal cord tumor had been completely removed, and no muscle damage of C3 had occurred at all. In this case, we could completely remove the spinal cord tumor without causing any muscle damage.

This is a case of a 66-year-old man with C2-C3 schwannoma. Preoperative images showed a spinal cord tumor extending from C2 to C3 (Figure 2). In this patient, we performed tumor removal with complete preservation of the C2 muscles, which is important for maintaining postoperative cervical alignment [9] (Figure 3).

To date, we have used this system to determine surgical methods for five spinal cord tumors. There were one cervical, two thoracic, and two lumbar cases. A laminectomy was performed in four cases and a laminoplasty in one case. In all cases, the tumors were completely removed with a less invasive technique, and the symptoms improved postoperatively (Table 1).

**Table 1:** Spinal cord tumor cases

	Age	Gender	Tumor site	Surgical method	Pathology
Case 1	63	Male	L3	L3 laminectomy	Schwannoma
Case 2	66	Male	C2-C3	C2-C3 laminoplasty	Schwannoma
Case 3	45	Male	Th12	Th11-12 laminectomy	Schwannoma
Case 4	66	Female	L1	L1 laminectomy	Schwannoma
Case 5	64	Male	Th10	Th9-10 laminectomy	Schwannoma

Technical note

**Figure 4:**
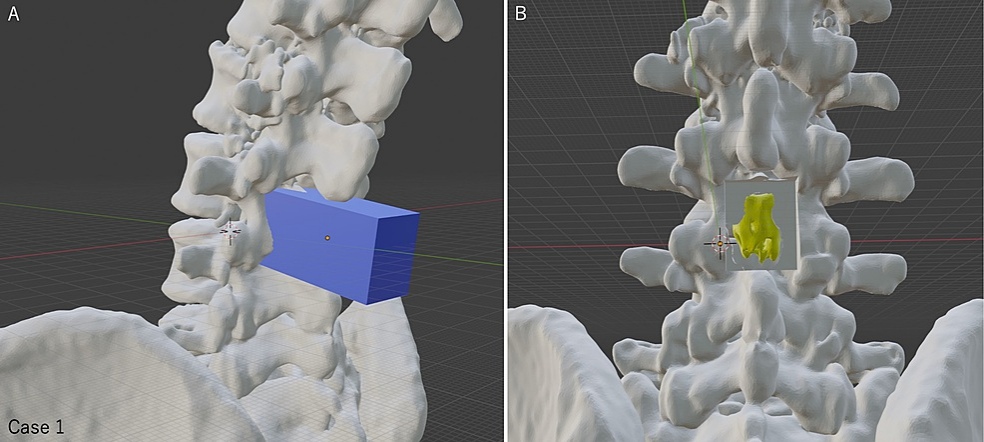
Making planned laminectomy model using the 3D editing software Blender (case 1) (A) We determined the site of the laminectomy and placed a rectangular cube over the bone resection site. This cube's length, width, and height can be set in millimeters. Bone regions that overlap with the rectangle can be removed from the original bone model. In this case, a cube with a width of 24 mm was used to perform a planned laminectomy. (B) The 3D model after L3 laminectomy showed the spinal cord tumor in the spinal canal. The tumor appeared to be safely removed with this planned width of laminectomy.

Myelogram-CT images were taken using Aquilion ONE Vision Edition (Canon Medical Systems Corporation, Tochigi, Japan) or Brilliance 64-CT scanner (Philips, Tokyo, Japan) to create 3D images for the spine and spinal cord tumors. These images were created using a workstation (Ziostation2 Plus, Ziosoft Corporation, Tokyo, Japan). When identifying the tumor was difficult, the myelogram-CT and MRI images were checked individually and fine-tuned to achieve the best position of the spinal cord tumor. The STL data of both bony elements and the spinal cord tumor were then output by a workstation. A free 3D image editing software (Blender, Blender Foundation, Amsterdam, the Netherlands) was applied to the STL data to create the laminectomy model. The planned decompression area of the bone element was deleted from the original spinal data using the Blender software (Figure 4, case 1).

**Figure 5:**
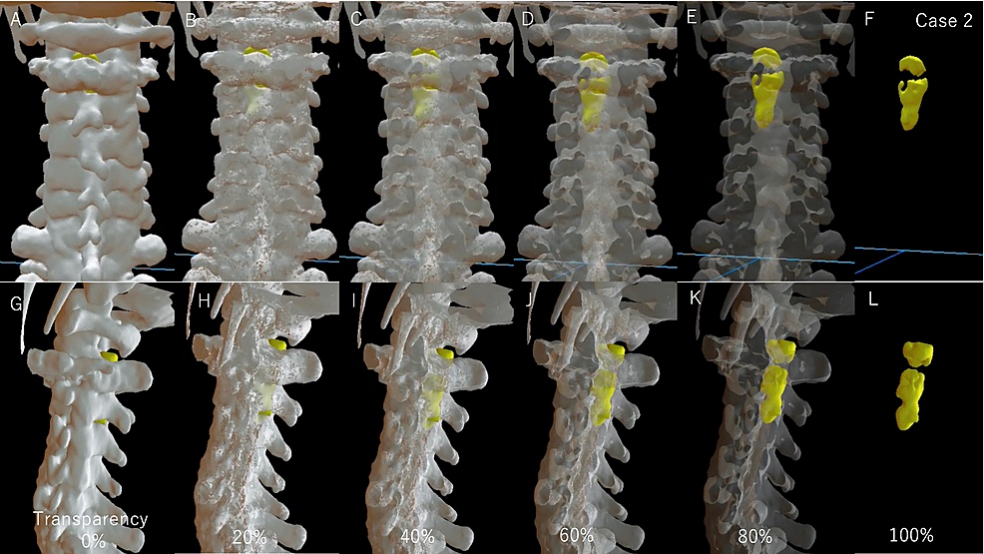
Differences in 3D model visualization due to changes in bone transparency (case 2) (A-F) The 3D model was seen from the dorsal side. (G-L) The 3D model was seen from the left side. A, G. Bone transparency was 0%. (B, H) Bone transparency was 20%. (C, I) Bone transparency was 40%. D, J) Bone transparency was 60%. (E, K) Bone transparency was 80%. (F, L) Bone transparency was 100%. Increased bone transparency makes it easier to see the spinal cord tumor. Seeing a full-scale spine and spinal cord tumor in 3D with an HMD can quickly grasp the positional relationship, and the surgical technique can be intuitively determined. HMD, head-mounted display

These three data from the original spinal bone, spinal cord tumor, and created laminectomy image are displayed on the HMD, MR devices (HoloLens 2, Microsoft Corporation, Redmond, WA, and Magic Leap 1, Magic Leap, Fort Lauderdale, FL) or virtual reality device (Oculus Quest; Facebook Technologies LLC, Menlo Park, CA), using a dedicated application (Holoeyes MD, Holoeyes Corporation, Tokyo Japan). In the Holoeyes MD software, the bone and tumor can be observed in three dimensions (Figure 5, case 2).

**Figure 6:**
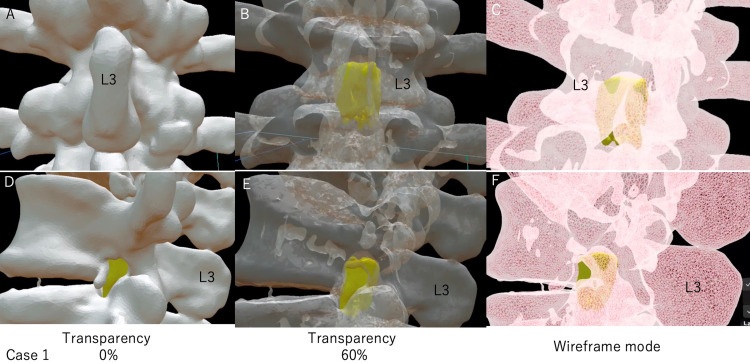
A 3D model image of the spinal cord tumor in the lumbar spine (case 1) Ten times magnification model was used to observe easily. We increased the spine transparency or used wireframe mode and made it easier to see the spinal cord tumor inside the spine. Observing these images with an HMD allows us to see the positional relationship directly in 3D and intuitively grasp it. (A-C) Dorsal view of the spinal cord tumor at the L3 level. The tumor was median and evenly distributed on both sides. We also confirmed little overlap between the L4 lamina and the spinal cord tumor. (D-F) The spinal cord tumor was seen from the left side. The tumor was located at the intervertebral disc level, and its anteroposterior diameter spans the entire spinal canal. The tumor was seen through the left intervertebral foramen. This spinal cord tumor seemed to be removed with only L3 laminectomy. (C, F) When we use wireframe mode for the spine, spinal cord tumors can be viewed from any angle without changing the bone transparency. This mode is advantageous when observing spinal cord tumors and also useful for determining the surgical site of bony elements because the bone shape can be seen more clearly than with the mode of increased transparency of the bone.

**Figure 7:**
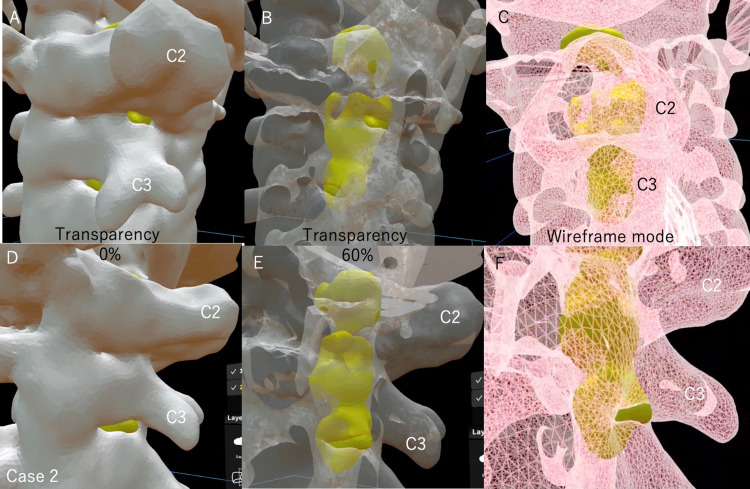
A 3D model image of the spinal cord tumor in the cervical spine (case 2) Ten times magnification model was used to observe easily. We increased the spine transparency or used wireframe mode and made it easier to see the spinal cord tumor inside the spine. Observing these images with an HMD allows us to see the positional relationship directly in 3D and intuitively grasp it. (A-C) Dorsal view of the spinal cord tumor at the C2-C3 level. The tumor was distributed on the left side. (D-F) The spinal cord tumor was seen from the left side. The spinal cord tumor extended the cephalad to the superior margin of the C2 lamina and caudal to the C4 lamina with a little overlap. This spinal cord tumor seemed to be removed with C2-C3 muscle-preserving laminoplasty. (C, F) When we use wireframe mode for the spine, spinal cord tumors can be viewed from any angle without changing the bone transparency. This mode is advantageous when observing spinal cord tumors and also useful for determining the surgical site of bony elements because the bone shape can be seen more clearly than with the mode of increased transparency of the bone.

With MR technology, this stereoscopic image is displayed as 3D as it is in the real body and can be fixed in place. Therefore, by displaying the stereoscopic data as true 3D using an HMD, the stereoscopic data can be intuitively grasped in the brain of surgeons. Holoeyes MD can arbitrarily display and hide each uploaded 3D image: bony elements, spinal cord tumor, planned laminectomy model, and other images including nerves and vessels. In addition, Holoeyes MD can adjust the transparency of each image (Figure 5) and magnify images up to 10 times for evaluators (Figures 6, 7).

**Figure 8:**
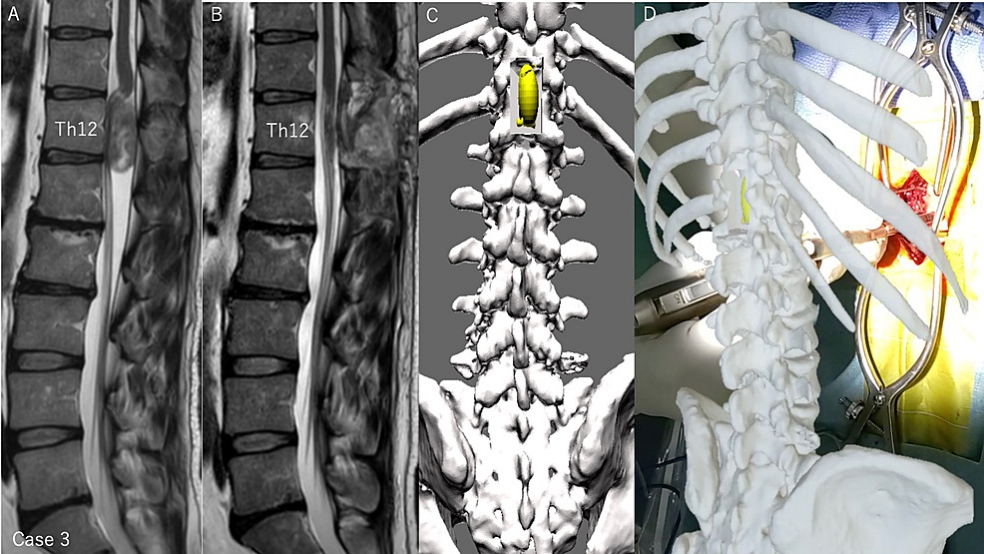
Pre- and postoperative MRI and 3D model of thoracic tumor case and intraoperative support with 3D model (case 3) (A) Preoperative MRI T2-weighted sagittal section showed a tumor including a cyst at the posterior part of Th12 vertebral body. (B) Postoperative MRI T2-weighted sagittal section showed no residual tumor and the laminectomy of Th11 caudal and Th12 lamina. (C) Dorsal view of the post-laminectomy model, in which the Th11 caudal and Th12 lamina were removed with a width of 20 mm. The margins of the spinal cord tumor can be seen, confirming that the tumor can be safely removed with this laminectomy. (D) Intraoperatively, the HMD could be used to confirm that the laminectomy was performed as planned in the surgical field.

We can observe these images from any angle at 360°. Spinal cord tumors can be easily seen transparently when the spine is viewed in wireframe mode (Figures 6, 7). The wireframe mode is useful for determining the surgical site of bony elements because the bone shape can be seen more clearly than with the mode of increased transparency of the bone. This system allows us to intuitively recognize the positional relationship between the tumor and the spine, making it easier to determine the surgical procedures. If we doubt the surgical site during the surgery, the 3D image can be overlaid on the patient's body through HMD of MR devices (Figure 8, case 3).

Results

Case 1: Lumbar Case

We were unsure whether to perform a partial L2 caudal laminectomy from the preoperative images (Figure 1). After observing the tumor location with HMD, preoperative 3D imaging evaluation revealed that L3 laminectomy alone was sufficient to remove the spinal cord tumor (Figure 6). In addition, this evaluation was confirmed preoperatively by creating a laminectomy model using the Blender software (Figure 4).

Case 2: Cervical Case

Due to the large size of the tumor based on the preoperative images, we considered a double door laminoplasty of C2 and laminectomy of C3. HMD showed that the tumor was localized to the left side, and hemilaminectomy seemed sufficient to remove the tumor at the C3 level. In addition, the extension of the tumor to the cephalad was limited to the cephalic margin of the C2 lamina, and it seemed possible to remove the tumor if it could be approached through half of the lamina of C2 and C3 (Figures 5, 7). Therefore, we decided to remove the tumor by C2 and C3 muscle-preserving laminoplasty.

## Discussion

Various methods have been attempted for the removal of spinal cord tumors [1-10]. There are several reports of the reduced amount of laminectomy and a small incision approach, leading to less blood loss and hospital stays [1-4]. Laminoplasty has been reported to be useful in preventing spinal deformity, preventing spinal fluid leakage, reducing bleeding, and shortening hospital stays [6,7,10]. Ultimately, the approach will depend on the type and localization of the tumor and the surgeon's ability to handle nerves and resect bones. A minimally invasive approach can always be helpful for patients if tumor removal can be performed safely and reliably [2,4,9]. Several reports compared spinal cord tumor removal techniques, but none of them clearly describes the appropriate site and level of indication for laminectomy or laminoplasty [2-4,10]. The introduced system is highly useful in terms of versatility because we can plan various surgical techniques [11]. This system can also be applied to intraoperative confirmation if necessary.

Using the Holoeyes MD software, it is possible to change the bone transparency and observe the tumor from any angle at 360°, which is very useful for planning spinal cord tumors removal. We could see the full-scale spine and tumor in front of us as if we were performing the spine surgery. Therefore, this system could intuitively suggest various surgical plans. In addition, the system can magnify the spine and tumor up to 10 times during the planning process, which is useful for observing fine details when we assess the margins of the tumor.

In our four cases of laminectomy, we had intuitively understood the extent of bone resection in the cephalocaudal and lateral directions preoperatively. In the case of laminoplasty, this system supported the decision to perform a less invasive surgery and visualized the tumor removal by laminoplasty with a preoperative prediction of the adequate location of the bony gutter.

## Conclusions

The MR system resulted in an intuitive determination of the surgical approach and spinal cord tumor removal area. In addition, by creating a laminectomy model using the 3D editing software Blender, we can predict and confirm the details of the tumor removal procedure before surgery. This planning allowed us to perform less invasive surgery with confidence. Furthermore, we could use it to identify the laminectomy site during surgery. This novel technique of the MR system can be useful to determine the surgical procedure for spinal cord tumors.
